# Supplementary material for: Identification of photoperiod-induced specific miRNAs in the adrenal glands of Sunite sheep (Ovis aries)
Source: Front Vet Sci. 2022 Jul 22;9:888207. doi: 10.3389/fvets.2022.888207 (PMC9354845; doi:10.3389/fvets.2022.888207)
Supplement: Supplementary file 1 [file Data_Sheet_1.ZIP › Supplementary file/miRDeep2 file.zip!/miRDeep2 file/Know_Structure/LP42A2/oar-mir-323a.pdf]

miRBase precursor : oar-mir-323a
Total read count : 1486
oar-miR-323a-5p read count : 11
oar-miR-323a-3p read count : 1472
remaining reads : 3

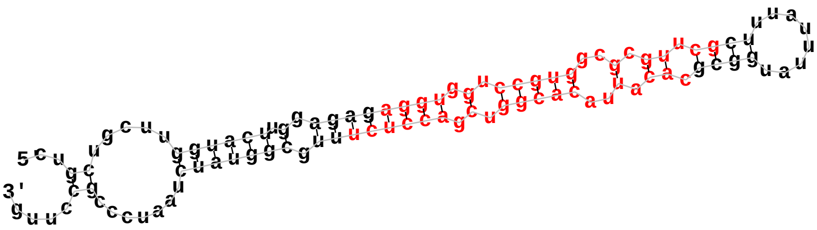

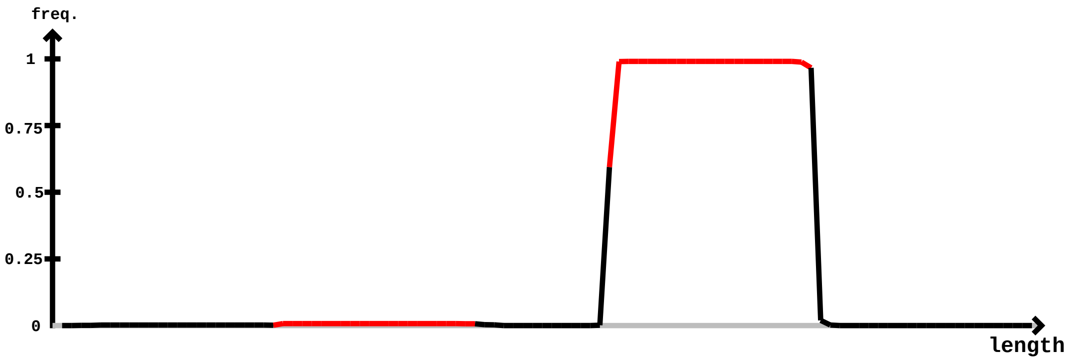


```
                                oar-miR-323a-5p
                                                                   oar-miR-323a-3p
5'- cugcugcuugguacuuggagagaggugguccguggcgcguucgcuuuauuuauggcgcacauuacacggucgaccucuuugcgguaucuaaucccgccuug   -3'    exp
    .........((((((.(.((((((((.(.(((((..(.((.((((........)))).)).)..))))).).)))))))).))))))).............    reads      mm         sample
    .ugcugcuugguacuuggagag...............................................................................    1          0          seq
    ...cugcuugguacuuggaga................................................................................    1          0          seq
    ...cugcuugguacuuggagag...............................................................................    1          0          seq
    ......................aggugguccguggcgcguu............................................................    1          0          seq
    ......................aggugguccguggcgcguucg..........................................................    5          0          seq
    ......................aggugguccguggcgcguucgc.........................................................    1          0          seq
    ......................aggugguccguggcgcguucgcu........................................................    4          0          seq
    .......................................................cgcacauuacacggucgaccuc........................    1          0          seq
    .......................................................cgcacauuacacggucgaccucu.......................    1          0          seq
    ........................................................gcacauuacacggucgaccu.........................    1          0          seq
    ........................................................gcacauuacacggucgaccuc........................    14         0          seq
    ........................................................gcacauuacacggucgaccucu.......................    850        0          seq
    ........................................................gcacauuacacggucgaccucuu......................    16         0          seq
    .........................................................cacauuacacggucgaccu.........................    3          0          seq
    .........................................................cacauuacacggucgaccuc........................    17         0          seq
    .........................................................cacauuacacggucgaccucu.......................    555        0          seq
    .........................................................cacauuacacggucgaccucuu......................    10         0          seq
    .........................................................cacauuacacggucgaccucuuu.....................    3          0          seq
    ..........................................................acauuacacggucgaccucu.......................    1          0          seq
```
